# Detection of Genomic Idiosyncrasies Using Fuzzy Phylogenetic Profiles

**DOI:** 10.1371/journal.pone.0052854

**Published:** 2013-01-14

**Authors:** Fotis E. Psomopoulos, Pericles A. Mitkas, Christos A. Ouzounis

**Affiliations:** 1 Department of Electrical and Computer Engineering, Aristotle University of Thessaloniki, Thessaloniki, Greece; 2 Centre for Bioinformatics, Department of Informatics, School of Natural and Mathematical Sciences, King’s College London, Strand, London, United Kingdom; University of Cyprus, Cyprus

## Abstract

Phylogenetic profiles express the presence or absence of genes and their homologs across a number of reference genomes. They have emerged as an elegant representation framework for comparative genomics and have been used for the genome-wide inference and discovery of functionally linked genes or metabolic pathways. As the number of reference genomes grows, there is an acute need for faster and more accurate methods for phylogenetic profile analysis with increased performance in speed and quality. We propose a novel, efficient method for the detection of genomic idiosyncrasies, i.e. sets of genes found in a specific genome with peculiar phylogenetic properties, such as intra-genome correlations or inter-genome relationships. Our algorithm is a four-step process where genome profiles are first defined as fuzzy vectors, then discretized to binary vectors, followed by a de-noising step, and finally a comparison step to generate intra- and inter-genome distances for each gene profile. The method is validated with a carefully selected benchmark set of five reference genomes, using a range of approaches regarding similarity metrics and pre-processing stages for noise reduction. We demonstrate that the fuzzy profile method consistently identifies the actual phylogenetic relationship and origin of the genes under consideration for the majority of the cases, while the detected outliers are found to be particular genes with peculiar phylogenetic patterns. The proposed method provides a time-efficient and highly scalable approach for phylogenetic stratification, with the detected groups of genes being either similar to their own genome profile or different from it, thus revealing atypical evolutionary histories.

## Introduction

Phylogenetic profiles are binary representations that record the presence or absence of a gene across a range of species [1]. Previous incarnations of this formalism had been proposed in terms of sequence pattern distributions across taxonomic domains [2]. Phylogenetic profiles have been used for the inference of function networks [1], along conserved gene clusters [3], [4] and gene fusions [5], [6], collectively known as genome context methods.

Evidently, the formulation of phylogenetic profiles can be generalized to record gene (or protein) families instead of single genes [2], [7], with various metrics expressing the presence of a cluster, and indeed across higher taxonomic categories [8]. Furthermore, similarity of profiles can be treated by probabilistic methods other than Hamming distance, including Pearson correlation coefficient and mutual information [9]. Despite the elegance of the approach, as well as its general and expandable character, phylogenetic profiling raises a number of conceptual and technical issues that have proven to be highly challenging.

First, the functional relationship signal is often masked by a strong evolutionary signal (i.e. highly similar, yet functionally unrelated genes have similar profiles); this issue is usually addressed by pre-processing similar genes and excluding them from further analysis, especially in the context of network inference [10]. Certain approaches towards this direction have been proposed, including automated error correction [11], the introduction of decision rules [12] and the use of weighted phylogenetic profiles according to a wide range of criteria [13].

Second, phylogenetic profile signals can be quite noisy, thus lowering the performance of the method for genome-wide function prediction. Multiple benchmarks of the entire set of genome context methods have been performed, strongly suggesting that phylogenetic profiles typically exhibit higher recall and lower precision than gene clusters or fusions, in that order [14]. These initial studies have been supplemented by more recent analyses [15], [16]. Various other groups have examined the role of statistical significance testing for improved performance [17], the effect of genome structure and redundancy [18], and the choice of similarity metrics and inferred network topologies [19].

Third, there are certain subtleties of biological nature for the choice of query and reference organisms. Eukaryotic genomes appear to perform less well than prokaryotic genomes as queries, possibly due to the presence of promiscuous protein domains and the narrower taxonomic range of the reference dataset [20]. The choice of the reference dataset obviously affects the outcome of network inference as well: the broader the range, the better the performance [21]. Calibration and control of these factors might be obtained by the use of genome trees and more robust phylogenies [7], [22] – that are less sensitive to effects such as horizontal gene transfer or gene loss than gene-based trees [23], [24] – or, more plainly, the mere collapse of highly similar genomes [12].

Finally, an interesting avenue of research has been the correlation of gene (phylogenetic) profiles with trait (phenotypic) profiles for the direct detection of genotype-phenotype associations [25], [26]. These phenotypes can include traits such as optimal growth temperature or pH [25] and oxygen dependence or motility [26]. While the results of these studies are encouraging, with the different approaches that have been followed, the biological interpretation of the findings on a genome-wide scale awaits a more thorough evaluation by independently derived data and future experimental verification. This is particularly crucial for phenotypes such as human diseases and their detected correlations with certain gene sets [27]. These associations have been generalized recently, by incorporating pathway profiles and their correlation with phenotypes, such as methanogenesis and other salient biochemical traits [28].

Recently, we proposed an approach based on the concept of ranked phylogenetic profiles and a benchmark dataset that addresses some of the issues above, especially the performance of the reference database [29]. In our quest for alternative representations, we now describe fuzzy profiles, with the aim to provide an efficient and scalable method for phylogenetic profile analysis, by reducing the initial noise of the query genomes and addressing certain additional limitations. Fuzzy profiles can thus detect genomic idiosyncrasies, by the direct comparison of individual gene profiles with the genome-wide profiles of the reference species. Some of these idiosyncratic traits might indeed correspond to sets of genes with evolutionary histories different from those of their source genomes.

## Methods

### Step 1: Creation of Fuzzy Phylogenetic Profiles

The use of fuzzy set theory in the life sciences has been reviewed elsewhere [30]. Following the fundamentals, the definition of a fuzzy genome phylogenetic profile is as follows. A species s_i_ is selected from a reference database of n species [i = 1.n] and a set of m_i_ phylogenetic profiles p_j_ [j = 1.m_i_], corresponding to the retrieved number of genes of species s_i_.

Each profile p_j_ is defined as a binary vector containing n values, i.e.
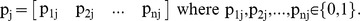



The fuzzy phylogenetic profile f_i_ of species s_i_ is defined as:
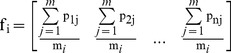
(1)


**Figure 1:**
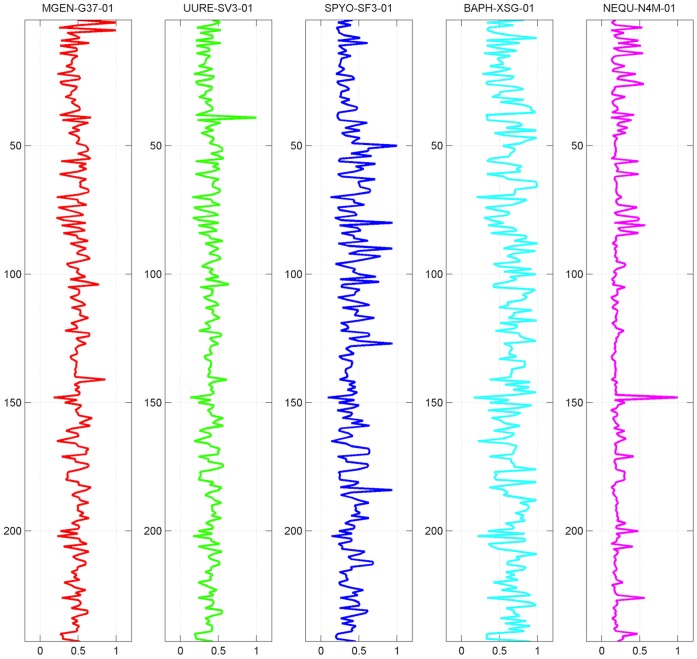
Fuzzy genome profiles for the five reference species used in this study (x-axis), against 243 species in the COGENT database (y-axis). The color-coding scheme for the five species is followed throughout all figures, where appropriate. Notice that the sequence of species ranks according to COGENT is #002, #039, #050, #088 and #148, reflected by the maximal values of the corresponding genome profiles.

The fuzzy phylogenetic profile is a real-value vector of n elements, as above (Equation 1). Each vector element in f_i’_ corresponds to the percentage of the genes in species s_i_ that are also present in species s_i’_ (or expressed, in case of expression) and thus represents a composite, ‘average’ behaviour of the total set of genes of the particular species. Genome profiles can thus be described as a summary of all gene profiles of a single species, each species being represented by a unique fuzzy genome profile. As a result, it is obviously expected that a vector element in f_i’_ corresponding to species s_i_ should be equal to 1 (**Figure 1**). In this study, we opted for a vertical representation, to distinguish fuzzy profiles from the more typical horizontal representation of gene profiles (**Figure 1**), while the maximal values of the genome profile are self-hits.

The next step is to calculate the distance between phylogenetic profiles of individual genes p_j_ and the genome profiles both of the same and different species f_i_. To achieve this, we need to define a pair of distance values, reflecting the distance measure of the individual gene profile against the same (intra-genome) and different (inter-genome) species, correspondingly, as follows:
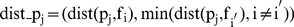
(2)where the first distance value clearly derives from the above definitions, while the second distance value is taken as the minimum of distances from all other reference species. This pair of distances essentially represents how different each gene profile p_j_ is compared to its source genome (intra-genome distance) and the closest reference species (minimum inter-genome distance – see also below, Step 4).

Besides the minimum function in Equation 2, other approaches could also be utilized, such as the arithmetic mean or a weighted function of all distances involved. In fact, the selected function is most appropriate for the given problem with regard to sensitivity (experiments with other measures not shown – see below for more information on the choice of distance metrics).

### Step 2: Discretization of Fuzzy Phylogenetic Profiles

To achieve a crisper clustering, the fuzzy profile f_i_ of a species might be transformed to a de-fuzzified one f_di_ (or an original, ‘digital’ profile, i.e. containing binary values, as opposed to ‘analog’, i.e. containing continuous values). This procedure can be performed as follows:
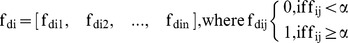
(3)


However, at this point we should consider the fact that phylogenetic profiles are known to have high noise levels, thus lowering their precision performance [14]–[16] (not shown). In order to compensate for this issue and increase the desirable contrast in the original phylogenetic data p_j_, an approach for dimensionality (and thus noise) reduction is needed.

### Step 3: Denoising of Phylogenetic Profiles with SVD

We have chosen to use Singular Value Decomposition (or SVD for short) [31], and apply it subsequently for the denoising of phylogenetic profiles p_j_ of the species under consideration. This, to our knowledge, is the first time that this approach has been used for the processing of phylogenetic profile data under the highly controlled conditions of a benchmark dataset [29] and on such a scale.

Given an m×n matrix A, whose rank is r, the eigenvalues of AA^T^ are:
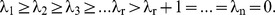




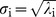
 is called singular value of A, where i = 1 … n.

Given an m×n matrix A, whose rank is r and m ≥ n, there exist two orthogonal matrices 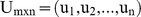 and 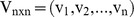 such that:
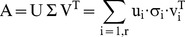
(4)


– where 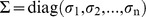 and σ_i_ is the singular value of A. Equation 4 is called the Singular Value Decomposition (SVD) of A.

By selecting the top k values σ_i_ of 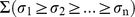 and setting the rest to 0, as part of the definition of SVD, we can construct an approximate representation of A.

It is evident that this “approximate” representation can be interpreted as “less noisy” regarding the particular case of phylogenetic profiles, as we demonstrate further in this study. The value of k can be selected by normalizing the values σ_ i_ between 0 and 1, and setting a coverage threshold λ, or SVD threshold. The values of σ_ i_ that add up to the coverage level λ (as a percentage), are a sufficiently accurate representation of the initial records at this coverage level. Consequently, the inverse transformation will yield a real-valued m×n matrix A’.

To map to the phylogenetic profile data, each row of matrix A corresponds to the profile p_j_ of a single gene; the transformed matrix retains this correspondence. In both cases, the number of rows of both matrices is equal to the number of input phylogenetic profiles.

In order to re-create a binary representation, an approximation would be to set any value larger than a specific threshold α to 1, and the rest to 0, according to Equation 3. Threshold α is therefore the key parameter by which the de-fuzzification process is achieved, with α representing the threshold cut-off value.

### Interlude: Definition of Distance Metrics between Two Vectors x_r_ and x_s_

We use the following definitions as distance metrics further in this study. The cosine distance measure is equivalent to one minus the cosine of the included angle between points (treated as vectors). Each centroid is the mean of the points in that cluster, after normalizing those points to unit Euclidean length.
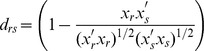



The Jaccard distance measure is equivalent to one minus the Jaccard coefficient, also used in this context previously [13], [20]. It represents the percentage of nonzero coordinates that differ.
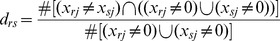



In practice the cosine metric is better suited for real-value vectors, and Jaccard distance has been shown to be better fitted for binary (discrete) vector distances [20].

These distance measures can be used for the comparison of each gene profile against any genome profile (according to Equation 2, in our case). As is evident from above, all profile data are now de-fuzzified; consequently, we generally opted to use Jaccard distance, after extensive comparisons. Since we do not perform an all-against-all profile comparison (where one could describe a clustering diagram capturing all profile-profile distance data), comparison of gene profiles against genome profiles only depends on the number of gene profiles in a linear fashion thus achieving the desired performance. The computed distance matrix capturing intra- and inter- genome relationships is defined as a ‘distance diagram’.

### Step 4: Determination of Profile Distances

**Figure 2:**
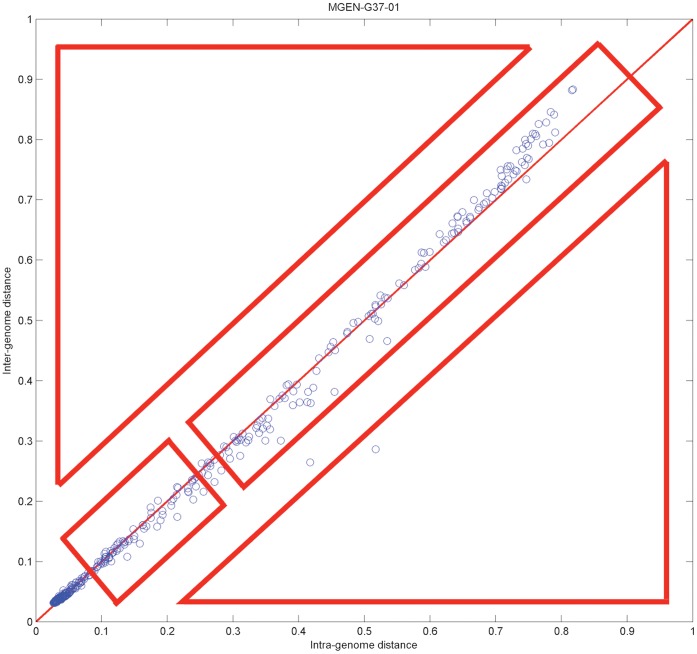
Example distance diagram, showing the four different areas of interest. The specific diagram is derived from *M. genitalium* as described, using the following parameters: no discretization process (both on fuzzy genome profiles and de-noized phylogenetic profile data – therefore, parameter alpha is not applicable); SVD threshold λ = 0.75; distance measure: cosine (default choice for real-value vectors). Evidently, most genes in this case are found close to the main diagonal; this might not be the case for other species.

Regardless of the actual distance metric used to detect the inter−/intra-genome distances, the actual metric of Equation 2 allows a precise user-defined quantity by which individual gene profiles can be compared against reference genomes (see also above, Step 1). By laying out all corresponding values on a two-dimensional graph with axes representing the source against the other reference genomes, it is possible to distinguish varying behaviours of individual genes against these backgrounds. In particular, the following areas can be evidently seen on the distance diagram of phylogenetic profiles (**Figure 2**).

This space can be decomposed into four areas:

- Lower left, on-diagonal: in this area, genes have low distance both in inter- and intra-genome comparisons. Typically, this area would contain genes that are common in all species.
- Upper right, on-diagonal: genes in this area have consistently increasing distance from both inter- and intra-genome comparisons.
- Upper left, off-diagonal: genes in this area have high inter-genomic and low intra-genomic distance. Typically, this area would cover genome-specific genes.
- Lower right, off-diagonal: genes in this area have low inter-genomic and high intra-genomic distance. Typically, this area would represent genes with unexpected phylogenetic/species distributions, occasionally deriving from external ‘donor’ species.

The latter areas (c, d) are located at the off-diagonal sections of this space and contain those genes with the least expected, ‘non-canonical’ behaviour with respect to their source genomes, according to the distance measures defined above. In other words, the application of the fuzzy profile method and the mapping of inter- and intra-genome differences on the distance diagram allow the detection of genome idiosyncrasies. This stratification of genes onto the four areas of the distance diagram with respect to the genome profiles thus reveals those genes with particular phylogenetic distribution and possibly different biological histories.

These genes are either highly genome/species-specific (as in the case of area c) or putative ‘foreign’ genes (as in the case of area d), both requiring further investigation to establish their origins.

**Figure 3:**
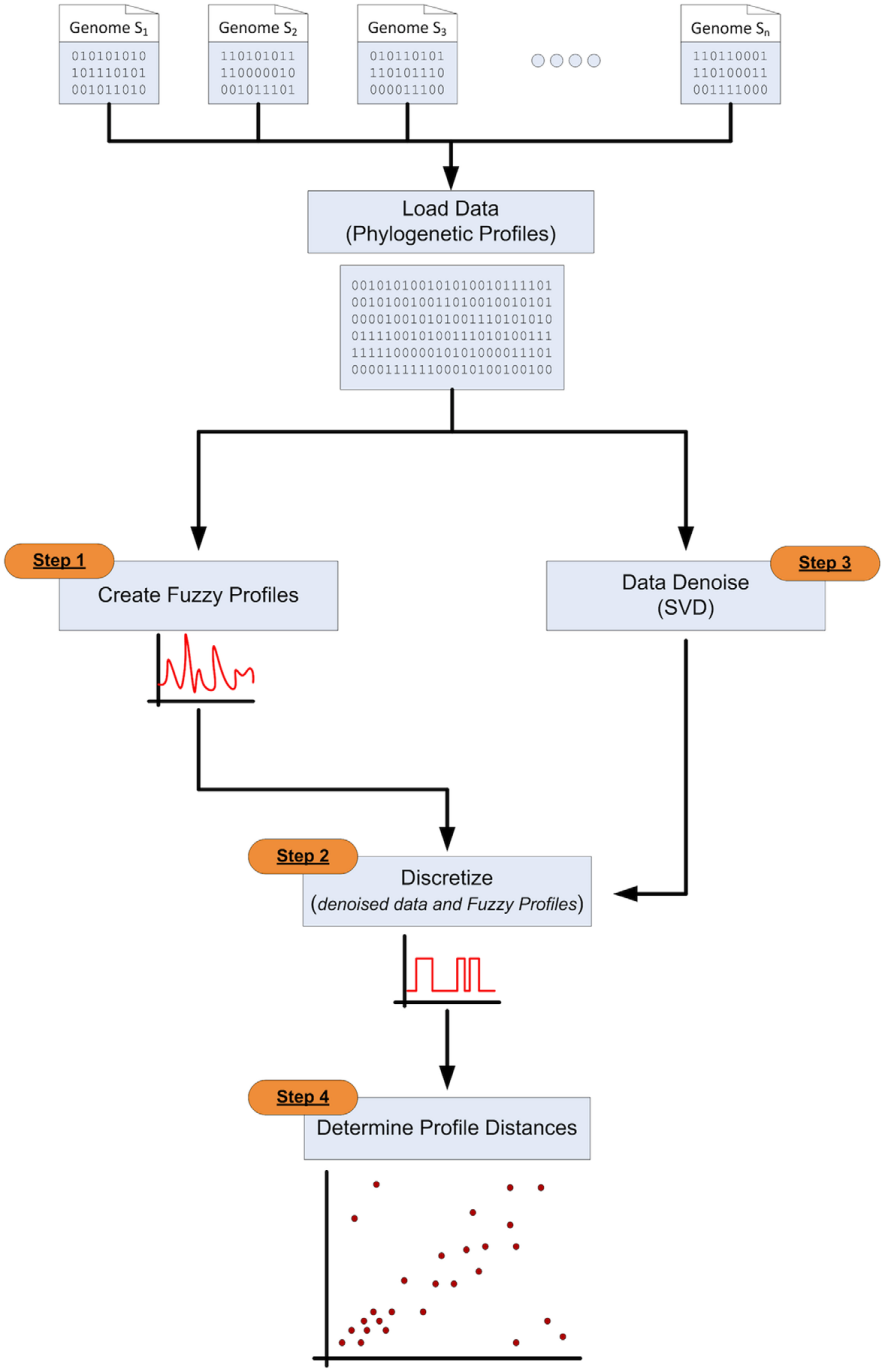
Flow diagram of the fuzzy profile method – see Methods for details.

The entire four-step process can be depicted as a sequence on a flow diagram, with the exception of the denoising step, which runs in parallel (**Figure 3** **)**.

We demonstrate the usefulness of fuzzy phylogenetic profiles for the detection of certain categories of genes with a few characteristic examples of off-diagonally distributed genes in this representation of genomic distance space (**Figure 2**).

### Data Resources and Algorithms

Development and analysis were performed using data from the ProfUse section of the COGENT++ environment [32], using the original COGENT genome entries [33]. The latest ProfUse version contains 243 species and 915,554 phylogenetic profiles; these profiles are generated by database searching against the COGENT collection as the target database. The 3,896 gene profiles for the five reference species are made available as data input (see below). For the five species selected, both the phylogenetic profiles and the genome conservation scores were generated as previously described [22]. Sequence matching and database cross-referencing was performed using MagicMatch [34]. Any other database, sequence-matching algorithm and phylogenetic profile dataset can replace the above, since the framework is generally applicable as implemented.

## Results

To establish the method and validate it through a number of experiments, we have selected five species with small genomes, starting with the smallest and incorporating other small-genome representative species with increasing phylogenetic distance from the same taxonomic family, phylum and higher taxa, as described elsewhere [29]. These five-species benchmark dataset was used to perform parameter optimization, in addition to algorithm development. Herein, we describe: (i) the establishment of the benchmark dataset and a number of jack-knife tests to obtain distance diagrams for the five species, (ii) parameter optimization, (iii) an analysis of 12 outlier genes for the smallest genome and (iv) report on a software package that can be used for larger-scale analyses and further experimentation by the community.

### Selection of the Five Reference Species

The 5 reference species used for the experiment process are the following:

1. *Mycoplasma genitalium*, G-37 [35] (Bacteria; Firmicutes; Mollicutes; Mycoplasmatales) 479 genes, COGENT code: MGEN-G37-01.
2. *Ureaplasma urealyticum*, serovar 3 [36] (Bacteria; Firmicutes; Mollicutes; Mycoplasmatales) 613 genes, COGENT code: UURE-SV3-01.
3. *Streptococcus pyogenes* M1, SF370 [37] (Bacteria; Firmicutes; Bacilli; Lactobacillales) 1696 genes, COGENT code: SPYO-SF3-01.
4. *Buchnera aphidicola*, SG [38] (Bacteria; Proteobacteria; Gamma-proteobacteria; Enterobacteriales) 545 genes, COGENT code: BAPH-XSG-01.
5. *Nanoarchaeum equitans*, Kin4-M [39] (Archaea; Nanoarchaeota) 563 genes, COGENT code: NEQU-N4M-01.

**Figure 4:**
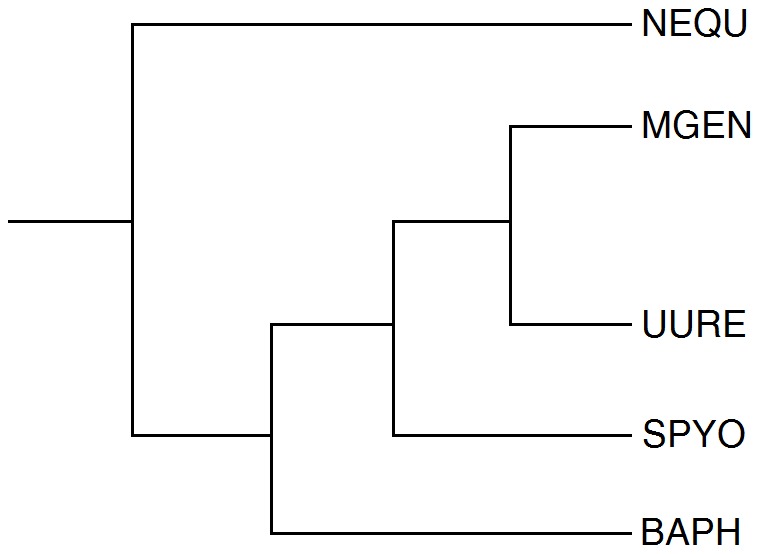
Simplified dendrogram representing the phylogenetic distances of the five reference species; COGENT species codes are used for brevity.

The total number of genes and corresponding profiles is 3,896. Code names are used interchangeably with the full strain name, or simply the species name (four-letter COGENT code pre-fix) in text, for brevity. A simplified dendrogram representing the phylogenetic relationships of the five species is shown in **Figure 4**. The full phylogenetic tree is provided in **File S1**.

**Figure 5:**
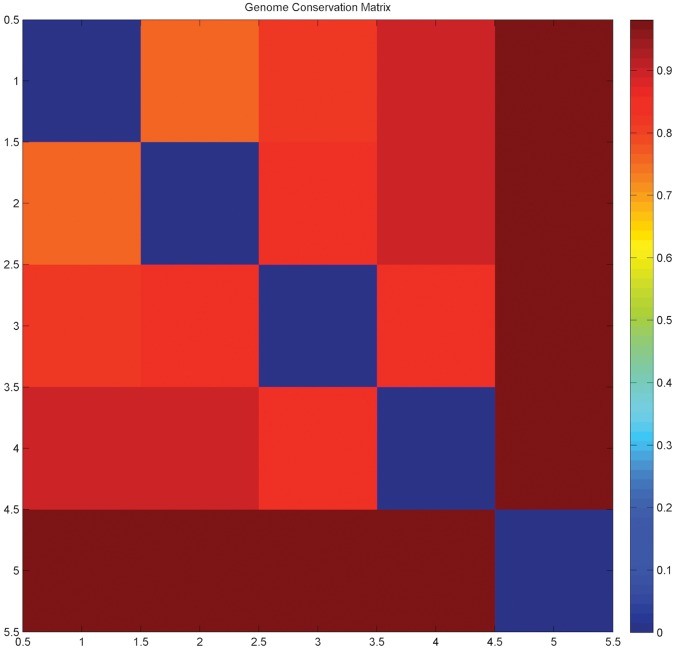
Distance matrix representing the distances between the five reference species, using the genome conservation metric which ranges between 0 and 1 (normalized values) [**22**]. The diagonal self-distance values are evidently zero.

Genome distances were obtained from a full genome comparison of 243 species [22]. The ‘genome conservation’ matrix containing the distances for the five species is provided in **Table 1** and visually in **Figure 5** **.** We regard the choice of reference species, with the above criteria outlined, as part of the experimental design supporting the proper validation of our method.

**Table 1:** Normalized phylogenetic distance values for the five reference species, pictorially shown in Figure 5.

	MGEN	UURE	SPYO	BAPH	NEQU
MGEN	0	0.7660	0.8250	0.8900	0.9740
UURE	0.7660	0	0.8500	0.9010	0.9810
SPYO	0.8250	0.8500	0	0.8300	0.9700
BAPH	0.8900	0.9010	0.8300	0	0.9750
NEQU	0.9740	0.9810	0.9700	0.9750	0

### Generation of Fuzzy Genome Profiles for the Reference Species

Following the process as described previously, the fuzzy genome profiles of the 5 species are shown in **Figure 1**.

It is important to observe that the differences between the fuzzy profiles are more pronounced when the corresponding species might be isolated (**Figure 1**), as measured by the actual phylogenetic distances (**Figures 4**, **5**), the most distant species being *N. equitans* (**Figure 1**). This observation clearly supports the validity of the methodological approach, by clearly highlighting the phylogenetic distance of a species in this novel graphical representation.

**Figure 6:**
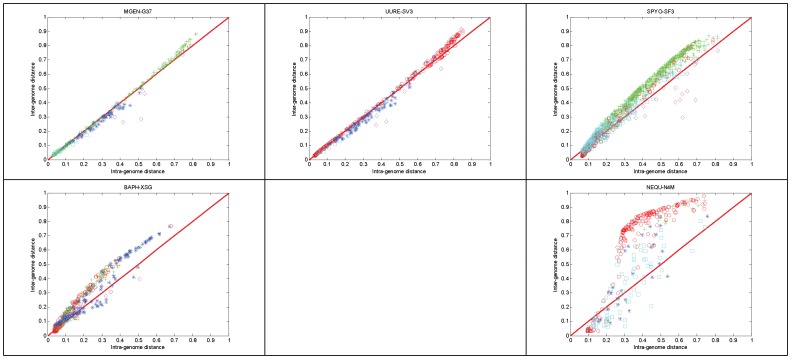
Distance diagrams of the 5 reference species. The upper-left panel representing *M. genitalium* is identical to **Figure 2**, except the color-coding scheme. This scheme encodes the genome profile of the species that produced the minimum inter-genome distance, as in **Figure 1**. Parameter settings as in **Figure 2**.

Using directly the genome fuzzy profiles as an ‘average’ representation of a genome for the gene phylogenetic profile comparison, and using cosine as the distance metric, the following distance diagrams can be produced (**Figure 6**).

Every gene is shown as a single point with the following coordinates: {distance from the source species, minimum distance from all other species}, in other words, {intra-genome distance, minimum inter-genome distance} (**Figure 6**). It is interesting to note that genes are primarily positioned along the main diagonal, in most cases, with notable exceptions (e.g. *N. equitans*). In the case of *M. genitalium* and *U. urealyticum*, there is a clear distribution of genes along the diagonals, thus signifying the affinity of the two species: for instance, in *M. genitalium*, most (*sic* typical) genes with either low intra- or inter-genome distance exhibit similarities to *S. pyogenes*, while the less typical genes (higher distances) are best related to *U. urealyticum* – similarly, the case is valid for the distance diagram of *U. urealyticum*, in a highly consistent fashion.

In the top three reference species distance diagrams, it is also evident that few genes exhibit lowest distance to *N. equitans*, as off-diagonal outliers (**Figure 6**). The most ‘unexpected’ behavior is indeed exhibited by the latter species, with no clear pattern emerging; this might be attributed partly to its distant phylogenetic position with respect to the other four reference species (**Figure 6**, lower right panel).

Overall, it can be argued that this novel representation demonstrates clearly, and in a comparative mode, that the method is able not only to delineate the differential phylogenetic context of the gene profiles in a biologically meaningful manner, but also stratify those genes within the distance space.

### Transformation of Fuzzy Phylogenetic Profiles to De-fuzzified Vectors

Despite the fact that the method is able to identify the source genomes in this particular representation of genome profiles (**Figure 1**), it is important to address issues of noise reduction and obtain a crisper representation, much resembling the original definition of phylogenetic profiles as binary vectors (**Methods**, Step 2). To achieve this, we control fuzziness with the parameter 〈 (Equation 3).

**Figure 7:**
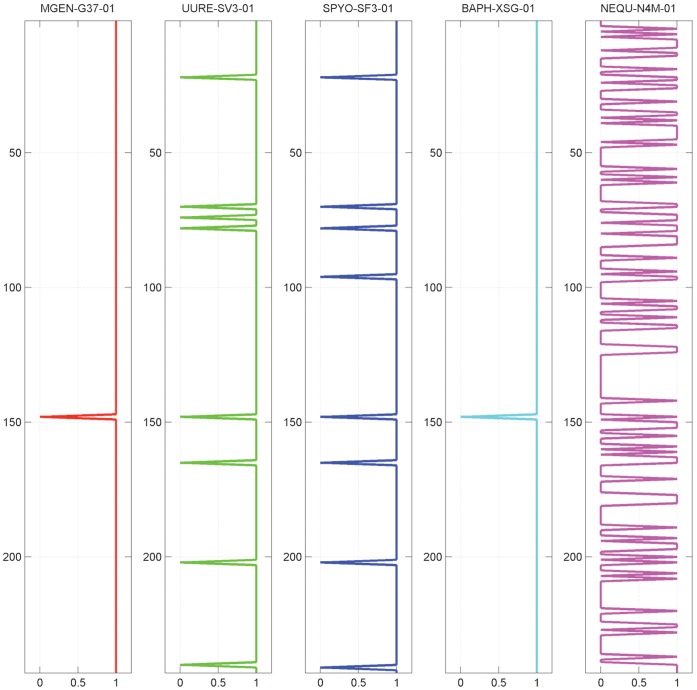
Discretized fuzzy genome profiles of the 5 reference species, using a low, permissive fuzzy threshold α = 0.2.

By setting a low, permissive threshold value α = 0.2, the fuzzy genome profiles are converted to ‘digital’ profiles, following the original binary representation. In this extreme case, the five genome profiles exhibit very high coverage of the database and demonstrate, once again, the ability of the method to also stratify entire genomes with respect to the target database content (**Figure 7**). In either case, with the analog or digital profile (**Figures 1**, **7**, respectively), the genome profiles identify their source genome as self-hits with varying degrees of success (the more permissive the easier, as in the present case).

Comparing species *B. aphidicola* and *N. equitans*, this analog-to-digital transformation is most pronounced (**Figure 7**). At the same time, it is possible to assess the target database ‘enrichment’ or over−/under-representation of a given species’ genome: *B. aphidicola* is evidently over-represented than *N. equitans*, obviously because of its relative phylogenetic position and the corresponding species composition of the target database. Finally, in all cases, the four other genomes are not able to identify *N. equitans* and a few other, apparently distant, species (**Figure 7**). Conversely, *N. equitans* shows a fairly uniform distribution of presence/absence of its entire genome profile, for the same reasons. The corresponding distance diagrams (cf. **Figure 6**) effectively produce no outliers, while most points lie on the main diagonal (not shown).

**Figure 8:**
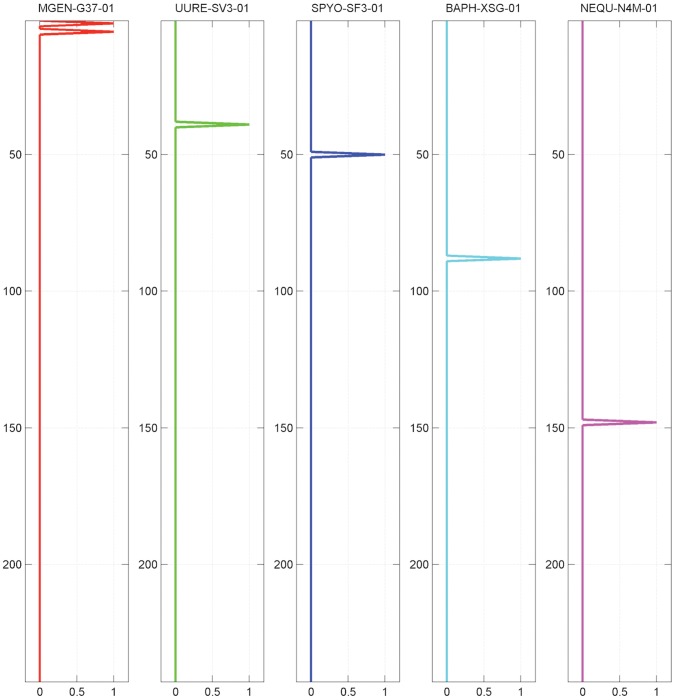
Discretized fuzzy genome profiles of the 5 reference species, using a high, stringent threshold α = 0.99.

At the other extreme of the de-fuzzification spectrum, with a high, stringent threshold value α = 0.99, the situation reverses: the ‘digital’ genome profiles essentially identify themselves as self-hits, against the target database. In this case, it is virtually impossible to assess the enrichment or over−/under-representation of the reference species against the entire data collection from which the profiles are generated (**Figure 8**). One minor exception is the ability of *M. genitalium* to identify *M. pneumoniae* (left panel, **Figure 8**): for those species, the conservation distance between them is 0.3080, whereas the minimum distance among the five reference species considered here is 0.7660 (**Table 1**).

By setting the highest value of α = 1, each genome profile recognizes only its source species: this uniquely flexible, parameter-driven representation provides the ability to conduct jack-knife tests as discussed above.

### Application of SVD Following Fuzzy Genome Profile Generation

After significant experimentation (see below), we therefore decided to perform validation experiments with the following parameter set:

- de-fuzzification threshold α = 0.35;
- SVD threshold λ = 0.75;
- Jaccard distance metric.

**Figure 9:**
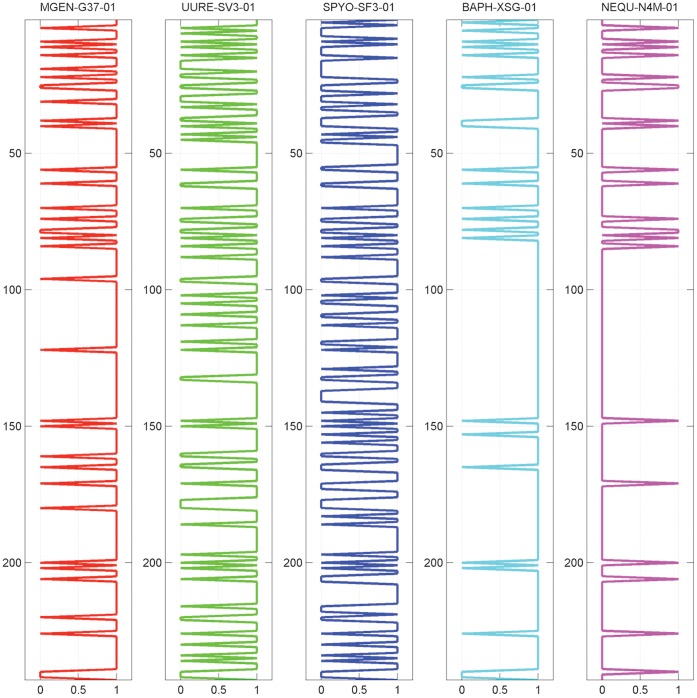
Discretized fuzzy genome profiles of the 5 reference species, using a fuzzy threshold α = 0.35.

As should follow from the above, the threshold 〈 represents a middle value between the two extreme scenarios, with sufficient database variability still maintained in the genome profiles (**Figure 9**). Concurrently, we perform the de-noising step with SVD, resulting in an approximate representation by setting a coverage threshold λ (see **Methods**) and measuring distance by the Jaccard metric.

**Figure 10:**
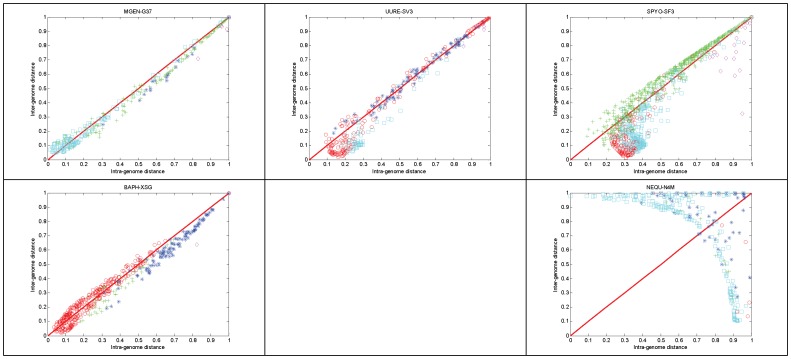
Distance diagrams of the 5 reference species, using the following parameters: fuzzy threshold α = 0.35; SVD threshold λ = 0.75; Jaccard distance metric. Corresponding fuzzy profiles are identical to those displayed in Figure 9 and color-coding as in Figure 6.

The distance diagrams for the five reference species chosen in this analysis are significantly different (**Figure 10**), reflecting the effects of the sensitive de-fuzzification threshold and the subsequent reconstruction of fuzzy profiles into binary profiles. The most pronounced differences are exhibited in *S. pyogenes* and *N. equitans*, where in the former case the distances are expanded due to threshold values, while in the latter case the distances are partioned into two off-diagonal groups with extreme inter- and intra-genome distance values (**Figure 10**).

It should be noted that we have chosen to use SVD for the denoising of the binary profile representation (**Figure 3**), as we have discovered empirically that performing this step on a fuzzy representation would create significant deviations from the original phylogenetic signals (not shown). In other words, if the fuzzy profiles are de-fuzzified, the use of SVD maintains data integrity.

### Search for Optimal Parameter Values

**Figure 11:**
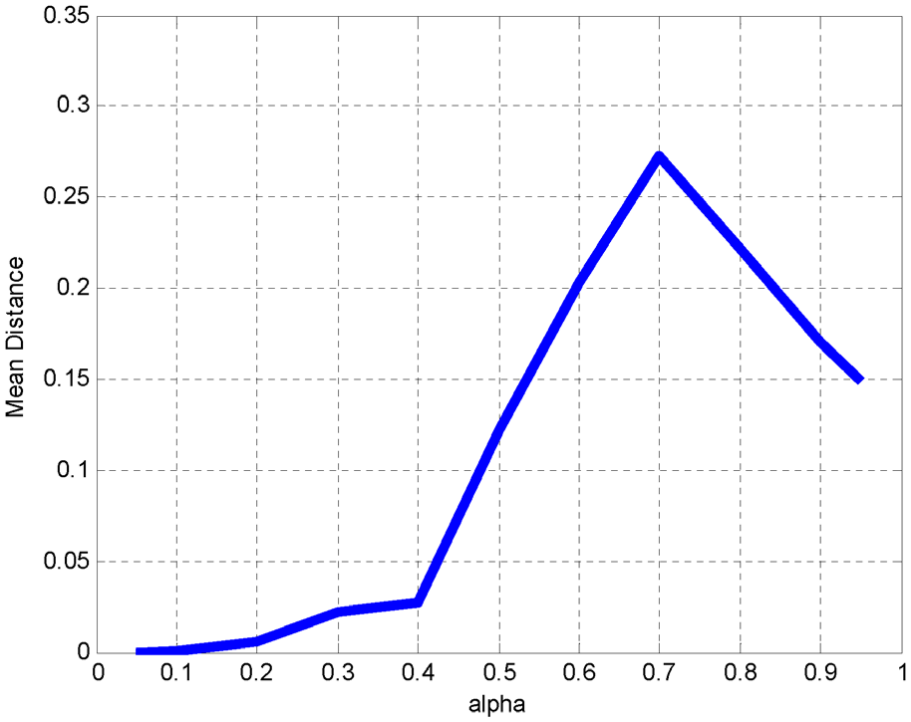
Parameter optimization for threshold α. By keeping parameters distance metric (Jaccard) and SVD threshold λ (0.75) constant, α is set to different values (x-axis). Distance distributions for all genes are derived from the main diagonal and within the distance diagram; mean distance is shown (y-axis). It is evident that there is an inflection point at α = 0.4 beyond which distances become sharply larger, thus indicating a higher disparity of gene profiles and a divergence from the expected presence of their corresponding coordinates along the main diagonal. This value can be taken as a maximal optimum value. Aiming at the most flexible value of α, without losing the on-diagonal presence of genes, an optimum range is between 0.3 and 0.4, hence the selection of 0.35 as our default α value.

Evidently, the approach of fuzzy phylogenetic profiles critically depends on the values of two numerical parameters namely α and λ, as well as the distance metric employed. We have seen above situations where extreme values of parameter 〈 are used and their effects on the jack-knife validation results **(** **Figures 7** **/8**), along with the optimal values we have chosen (**Figure 9**). To further justify the choice of parameters, we also provide the full scope of value exploration along the two numerical parameters and the distance metric (**File S2**). Optimal values are selected with respect to the mean distance of all points from the main diagonal, and the transitioning of these lower/higher, optimal mean distance values to higher/lower values assessed empirically by the choice of ‘inflection’ points of these curves (**File S2**, and example in **Figure 11**).

In this case, we choose as an optimal value of parameter α = 0.35, just before the mean distance curve climbs to higher values with α >0.40 (**Figure 11**).

### Biological Validation of Selected Cases

**Figure 12:**
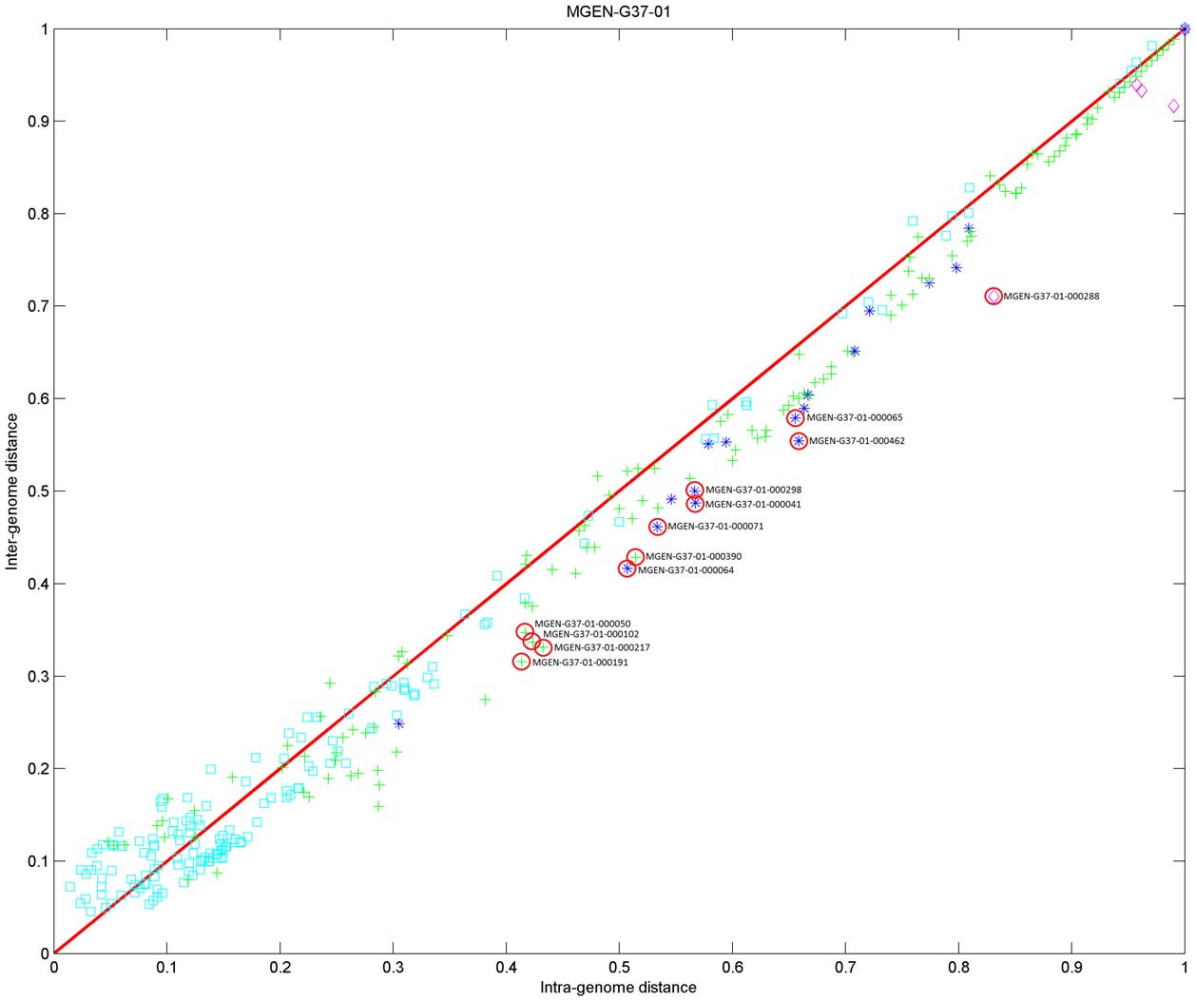
Distance diagram for *M. genitalium*, with the twelve outlier genes highlighted (see also **Table 2**). This diagram corresponds to the upper-left panel of Figure 10, with the same parameter settings.

To further validate the approach beyond the technical matters and the implicit jack-knife tests during the parameter search, we have decided to explore in more detail twelve outliers from the *M. genitalium* genome. These outliers are detected according to our method at the lower-right off-diagonal area of the distance matrix, with the following criteria for the Jaccard distance metric: i) intra-genome distance ≥0.4, and ii) intra−/inter-genome distance ratio ≥1.13, indicating a low inter-genomic and high intra-genomic distance (see above) and thus atypical evolutionary histories (**Figure 12**). Note that the latter does not necessarily imply horizontal gene transfer (HGT), although for half of the cases there is substantial evidence to support HGT (**Table 2**). We conclude that the fuzzy profile method is able to detect certain instances of HGT and other unusual phylogenetic distributions, under the criteria employed here. Note that the choice of outliers might vary according to the criteria set by users and the biological properties of the system under investigation: one could decide to extend the range of intra−/inter-genome distance values (**Table 2**) or, reversely, restrict them to capture a more limited set of outliers.

**Table 2:** Twelve cases selected from the *M. genitalium* genome according to specified Jaccard distance metric cut-off values (see text).

COGENT ID	ID§	Intra-genomedist §§	Inter-genomedist	Function	Taxa withhomologs	Comments
MGEN-G37-01-000288	MG283	0.8313	0.7105	prolyl-tRNA synthetase (ProS)	Mollicutes, Firmicutes, *Prevotella*	Belongs to the ProRS class II aaRS (present only in some bacteria), archaeal/eukaryotic type
MGEN-G37-01-000462	MG454	0.6587	0.5541	was: conserved hypothetical protein, Ohr/OsmC [41]	*mostly Proteobacteria (Shewanella, Vibrio, Photobacterium), Bacili (Enterococcus)*, Actinomycetales	Unique in *M. genitalium*, absent in *M. hominis* & *U. parvum*, as case MG062
MGEN-G37-01-000065	MG063	0.6555	0.5789	1-phosphofructokinase (FruK)	Mollicutes, Firmicutes, Fervidobacterium, Fusobacteriaceae, some Proteobacteria	Unique in *M. genitalium*, absent in *M. hominis* & *U. parvum*, as case MG062
MGEN-G37-01-000041	MG041	0.5673	0.4870	phosphocarrier protein HPr	Mollicutes, Firmicutes, *Thermotoga* and *Bacteroides*	Absent in *M. hominis*, present in *U. parvum* [42]
MGEN-G37-01-000298	MG293	0.5668	0.5000	glycerophosphoryl diester phosphodiesterase (GlpQ)	Mollicutes, Firmicutes, Thermoproteaceae	Unique in *M. genitalium*, absent in *M. hominis* & *U. parvum*, as case MG062
MGEN-G37-01-000071	MG069	0.5337	0.4615	putative PTS system glucose-specific EIICBA component (PstG)	Mollicutes, Firmicutes	Unique in *M. genitalium*, absent in *M. hominis* & *U. parvum*, as case MG062
MGEN-G37-01-000390	MG380	0.5144	0.4286	glucose-inhibited division protein B (GidB)	Mollicutes, Firmicutes, Spirochaetales, Thermotogaceae, some Proteobacteria	Somewhat dispersed phylogenetic distribution, Hydrogenothermaceae
MGEN-G37-01-000064	MG062	0.5072	0.4167	fructose-permease IIBC component (FruA)	Mollicutes, Firmicutes	Unique in *M. genitalium*, absent in *M. hominis* & *U. parvum* [42]
MGEN-G37-01-000217	MG214	0.4327	0.3314	conserved hypothetical protein	Mollicutes, Firmicutes	Similarity to a gene from *Ktedonobacter racemifer*
MGEN-G37-01-000192	MG189	0.4234	0.3368	ABC transporter (UgpE?)	Mollicutes, Firmicutes, Actinobacteridae	As case MG188
MGEN-G37-01-000050	MG050	0.4170	0.3472	deoxyribose-phosphate aldolase (DeoC)	Mollicutes, Firmicutes, Flavobacteriales and some Proteobacteria	Somewhat dispersed phylogenetic distribution, similar to orthologs from *Dictyoglomus* sp.
MGEN-G37-01-000191	MG188	0.4136	0.3155	ABC transporter (UgpA?)	Mollicutes, Firmicutes	Highly similar to group, glycerol transport

Both values have been experimentally validated to yield the maximum number of genes with respect to the trend across the main diagonal (Figure 12). Column names: COGENT identifier, common identifier (ID), intra-genome and inter-genome distances, described function, taxonomic categories (taxa) with homologs of corresponding genes and comments. The twelve cases are sorted by intra-genome distance in descending order, highlighting genes with the most anomalous phylogenetic distribution first.

§ putative cases of HGT are marked as **bold** in the ID column; remaining cases are classified into the Ugp/Glp and Fru/Pst groups;

§§ sorted by intra-genome distance.

### Biological Validation of the *M. genitalium* Genome Outliers

The phylogenetic profile outliers from *M. genitalium* are listed in **Table 2**. Of these, there are reasons to believe that MG050 might be a case of somewhat anomalous phylogenetic distribution indicating HGT [40]. Similarly, genes MG214, MG380 (GidB), MG041 (Hpr), MG454 (Ohr/OsmC [41]) and MG283 (ProS – from: http://bioinfo.mbb.yale.edu/genome/MG/extra/merge.db) are most likely cases of HGT, listed here with increasing intra-genome distance values (**Table 2**). More subtle cases are the group of genes MG062, MG063 and MG069, members of the fructose/glucose phosphoenolpyruvate-dependent sugar phosphotransferase transport system (PTS) and exclusively present in *M. genitalium* compared to other species of the group, including *M. hominis* and *U. parvum* [42]. The case of group containing genes MG188/MG189 and MG293 is less clear, encoding two ABC transporters and the glycerophosphoryl diester phosphodiesterase GlpQ, all parts of glycerol transport and metabolism. In all, under the defined criteria, we are able to detect 12 cases of putative exogenous genes in *M. genitalium*, a number comparable with the (possibly over-estimated) 50 or so genes detected as potential HGT cases solely based on base composition [43].

### Method Availability

We provide the entire module written in MATLAB and sufficiently documented along with sample input data for further experimentation by the community, as **File S3**. We have performed analyses with various datasets of up to 20,000 profiles in <2 minutes on a typical workstation, with virtually linear performance (not shown).

## Discussion

The method presented here is demonstrated to be consistent with the phylogenetic relation and position of the genes involved, within a carefully chosen, highly controlled benchmark dataset [29]. Thus, fuzzy phylogenetic profiles primarily address issues of performance and noise reduction [20], delineating the evolutionary signal in genome-wide profile information. Singular value decomposition (SVD) is utilized to increase the contrast function within initial phylogenetic profile datasets. The parameters used have been extensively explored: the SVD step does not affect discrete (binary) genome-wide profile generation; the corresponding threshold parameter λ affects continuous genome-wide profiles, with significantly less impact than the de-fuzzification parameter α.

This approach presupposes the availability of a well-organized database such as COGENT [33], so that issues of pre-processing, ranking and validation are alleviated. For example, the generation of genome trees [22] can assist during the pre-processing stage as well as the definition of query and reference genomes [21]. The full sampling of phylogenetic datasets with deterministic approaches for noise reduction eliminates the need for statistical analysis and other stochastic treatment [17]. Moreover, our approach is independent of the ranking order of database entries [13], both at the level of phylogenetic profiles and reference species (i.e. genome sequences).

Comparison of fuzzy profiles with other methods based on statistics or ranked profiles indeed represents a highly interesting avenue for future analysis, but it is clearly beyond the scope of the present work. One limitation of the present method is its exact nature, requiring from users to design analyses carefully; it is not a data mining approach that returns the most prominent features in any type of analysis: instead, the query dataset must be crafted in a selective fashion.

### Conclusions

Overall, the method is demonstrated to be extremely efficient, both in terms of computational complexity and high scalability. Moreover, it can be used as a validation approach for further studies, including correlation with phenotypic information [25], metagenomics datasets or metabolic pathways. In the near future, we intend to explore the phylogenetic profile formalism for a wider range of genomes and metagenomes as well as compare its performance with ranked profiles [29].

Indeed, the methodology can be used as a pre-processing step for several layers of genome analysis, including for instance the detection of atypical genes and other genomic idiosyncrasies. On the intra-genome level, the method can be utilized to identify single genes that exhibit interesting, species- or genome-specific traits. On the inter-genome level, whole genome collections can be evaluated for phylogenetic correlation of outlier genes, potential candidates of HGT. Ultimately, on the meta-genomic level, the methodology can be used with metagenomic sets as queries against genome collections for the detection of evolutionary and functional relationships.

## Supporting Information

File S1 **Full tree of five reference species.** Full tree of five reference species.
(BMP)

File S2 **Search for optimal parameters.** Optimization of parameter values for parameters α, λ and distance metric.
(PDF)

File S3 **Software.** Software application and documentation.
(GZ) Click here for additional data file.
