# Supplementary material for: Detection of Genomic Idiosyncrasies Using Fuzzy Phylogenetic Profiles
Source: PLoS One. 2013 Jan 14;8(1):e52854. doi: 10.1371/journal.pone.0052854 (PMC3544837; doi:10.1371/journal.pone.0052854)
Supplement: File S2 — Search for optimal parameters. Optimization of parameter values for parameters α, λ and distance metric. (PDF) [file pone.0052854.s002.pdf]

Parameter: α

α = 0.1	Distance Distribution
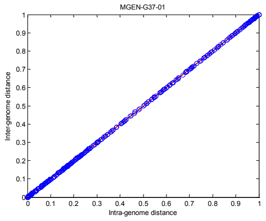	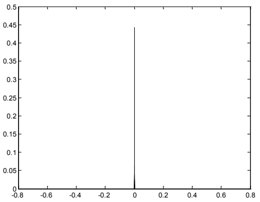
α = 0.3	Distance Distribution
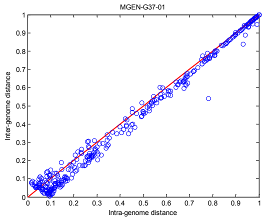	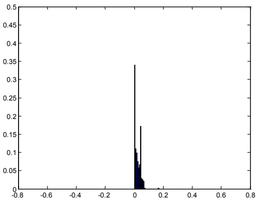
α = 0.6	Distance Distribution
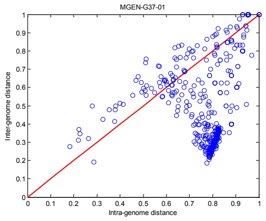	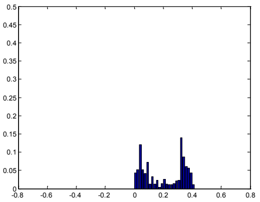
α = 0.9	Distance Distribution
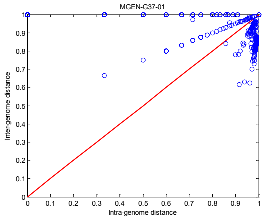	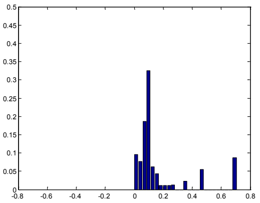

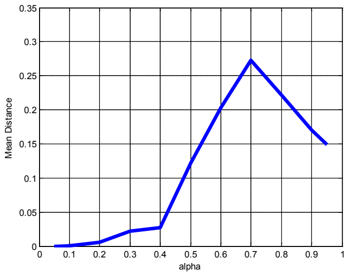

All Figures have been produced by keeping the parameters distance metric (*jaccard*) and SVD threshold λ (*0.75*) constant, and using different values for fuzzy threshold α. From the top figures, it is evident that the higher, more stringent values of α lead to a higher disparity of genes across the Distance Matrix; the expected presence of points on the main diagonal is lost. This is also evident by the average distance of each point from the main diagonal (bottom figure), where there is a sharp increase after 0.4. Therefore, aiming at the most flexible value of α, but without losing the on-diagonal presence of genes, the optimum range is between 0.3 and 0.4, hence the selection of 0.35 as our default α value.

Parameter: λ

λ = 0.1 | Distance Distribution

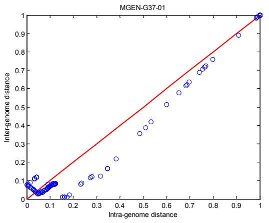

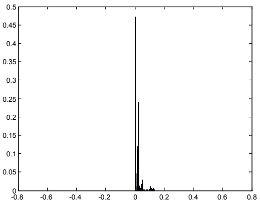

λ = 0.3 | Distance Distribution

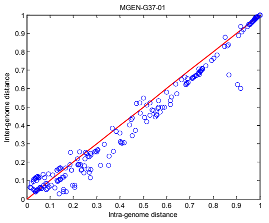

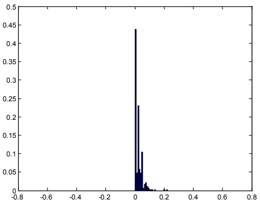

λ = 0.6 | Distance Distribution

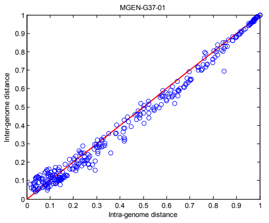

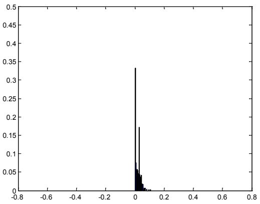

λ = 0.9 | Distance Distribution

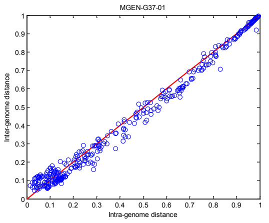

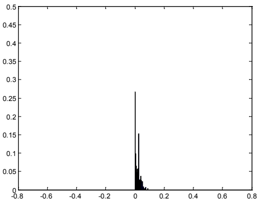

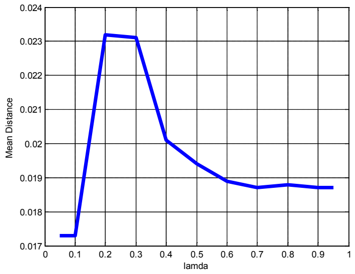

All Figures have been produced by keeping the parameters distance metric (*jaccard*) and fuzzy threshold α (*0.35*) constant, and using different values for SVD threshold λ. From the top figures it is evident that the lower values of λ (i.e. low percentage of representation of the initial records) severely modify the expected distribution of points on the Distance matrix. This is also evident by the average distance of each point from the main diagonal (bottom figure), where the mean off-diagonal distance reaches a steady state around the range of 0.7 to 0.9, hence the selection of 0.75 as our default λ value (we need the lowest possible λ value in order to maximize the de-noising effect but also retain the distribution of the original records).

Parameter: Distance metric

Metric: jaccard / Discretize: True	Distance Distribution
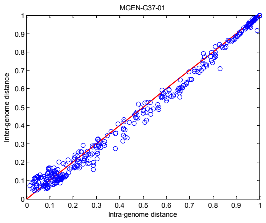	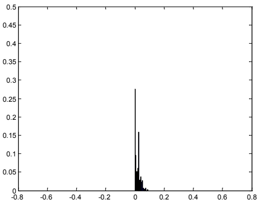
**Metric: cosine / Discretize: True**	**Distance Distribution**
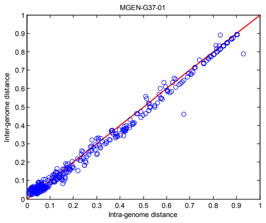	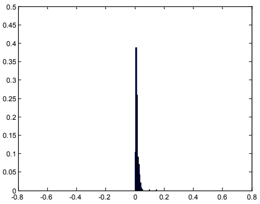
**Metric: jaccard / Discretize: False**	**Distance Distribution**
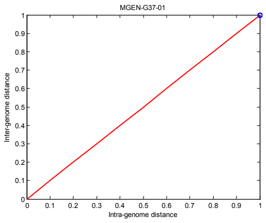	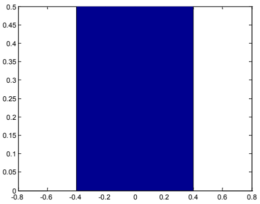
**Metric: cosine / Discretize: False**	**Distance Distribution**
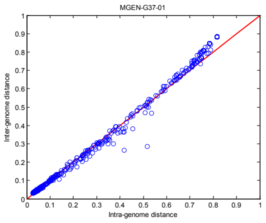	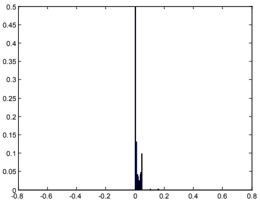

	**Jaccard**	**Cosine**
**Discretize: *True***	0.0188	0.0149 (*with NaN*)
**Discretize: *False***	0	0.0137

Average Distance. Green color denotes optimal selection; the opposite is shown in red color.

All Figures have been produced by keeping the parameters fuzzy threshold α (*0.35*) and SVD threshold λ (*0.75*) constant, and using different values for the distance metric. The top figures, together with the summarizing table in the bottom, validate the fact the Jaccard metric is only applicable on binary data (it is completely erroneous on the real-value data), whereas the Cosine metric is optimal for real-value data and quite ill-suited for the binary data (it can provide results but with a fair amount of NaN values).
